# Supplementary material for: Research on the impact of digital economy on labor resource allocation: Evidence from China
Source: PLoS One. 2024 Apr 17;19(4):e0297449. doi: 10.1371/journal.pone.0297449 (PMC11023257; doi:10.1371/journal.pone.0297449)
Supplement: Attachment — Submitted filename: Comments and Suggestions(PONE-D-23-09598).docx [file pone.0297449.s003.docx]

**Comments and Suggestions for Authors**

This paper focuses on the Impact of Digital Economy on Labor Resource Allocation. The topic is of practical significance. However, the level of this version needs to be improved, and the research logic and theoretical logic are not clear enough. It is not recommended to accept this version. It is suggested that the manuscript should be major revised and reviewed again. Some suggestions and comments are as follows：

1.The introduction is not focused enough and the logic is a little confused. As an international journal, it faces readers all over the world. First of all, the introduction needs to tell the reader why to study the allocation of labor resources and why to put the digital economy and labor resources into the unified framework for research. Secondly, summarize what research has been done on this topic, what are the shortcomings of these studies, and whether this paper makes up for these gaps. Thirdly, explain why the research is based on the reality of China and from which aspects this paper plans to carry out the research. Finally, the innovation of this paper is described.

2. Literature review is not comprehensive enough, there is no hierarchy and logic. For example, we can analyze it from the macro and micro perspectives or from the positive and negative effects of digital economy on the efficiency of labor resource allocation. Literature review is an in-depth and comprehensive review of a field. First of all, through literature review, readers can quickly have a general and clear understanding of your field of study. Secondly, through the literature review, it is found that there are some shortcomings in the existing literature, which can be made up for in your article.

3.Theoretical analysis lacks theoretical support. According to the content of the article, the author can make a theoretical analysis from the theory of resource allocation or the theory of information asymmetry. Theoretical analysis lacks theoretical logic, and many concluding arguments are not supported by literature and theory. For example：” These new features not only change labor relations but also profoundly affect the subjective perception of laborers, and reconstruct the evaluation system of labor resource allocation”. “The data-driven nature of the digital economy is characterized by the efficient presentation and dissemination of massive amounts of information, with data resources becoming a key driver of labor resource allocation. However, it can also create the problem of the digital divide…… cultivates a system of mass innovation and entrepreneurship, achieving flexible employment for many workers.” The whole paragraph is not supported by a single theory or literature. In addition, the analysis of the three paths is not clear enough, and it is suggested that the influence channels should be shown directly by drawing.

4.The empirical results are lack of comparative analysis and discussion. For example, it shows that the conclusion of this paper is different from the existing research, or the conclusion of this paper is consistent with some theoretical viewpoints.

5.At the bottom of each table, you need to note whether the empirical results are robust standard errors, and whether the parentheses in the table are T-values or variances.

6. In the conclusion part, first of all, we need to put forward targeted countermeasures against the negative impact of the development of digital economy. Secondly, the conclusion drawn from the heterogeneity part of this paper, there are no corresponding countermeasures and suggestions.

Finally, it is necessary to increase the shortcomings and future prospects of the study.

7. Most of the literature in this article is Chinese literature, which may lead to incomplete literature carding. It is suggested that the existing foreign literature should be combed carefully.

8．It is recommended to carefully check the expression and spelling in the text. For example, in the analysis in Table 5. Should digital economy (la) be changed from (la) to (de) in parentheses? there are three similar doubts, which are recommended to be examined carefully.
