# Supplementary material for: Research on the impact of digital economy on labor resource allocation: Evidence from China
Source: PLoS One. 2024 Apr 17;19(4):e0297449. doi: 10.1371/journal.pone.0297449 (PMC11023257; doi:10.1371/journal.pone.0297449)
Supplement: Attachment — Submitted filename: Response to Reviewers.docx [file pone.0297449.s004.docx]

Response to reviewer #1:

1.According to the literature by Wen Yanbing et al. (2022), both the 'Latin American trap' and the 'middle-income trap' are closely related to productivity losses caused by resource allocation. Additionally, the development of the digital economy provides the technological conditions for optimizing the allocation of labor resources. These factors serve as reasons to investigate the relationship between the digital economy and the allocation of labor resources. Furthermore, in order to enhance the persuasiveness of the introduction section, this paper includes information on the development of the digital economy in China. For instance,

“Digital economy has entered a new stage of rapid development in China and has become an important engine for the transformation of old and new drivers of growth as well as for promoting high-quality economic development. According to the "China Digital Economy Development White Paper (2021)" released by the China Academy of Information and Communications Technology, the scale of China's digital economy reached 39.2 trillion yuan in 2020, accounting for a high proportion of 38.6% of the GDP. The digital economy penetration rate in the service sector was 40.7%, far higher than the 21% in the industrial sector and 8.9% in the agricultural sector. Therefore, the digital economy has profound implications for factor markets, especially the labor market.”

Regarding the limitations of existing research, this paper addresses them in the literature review section by stating, ' based on previous research by scholars, this paper studies the subjective evaluation of Chinese workers on the relationship between the digital economy and labor resource allocation, in order to improve existing research.' Existing studies have focused on the impact of the digital economy on labor demand and structure, while lacking a subjective evaluation of the relationship between the digital economy and labor resource allocation.

China has carried out extensive practices in the development of the digital economy. As a developing country, its practices in digital economy development hold significant practical significance. Therefore, this paper starts from the reality in China and considers the subjective evaluation of the relationship between Chinese workers and the digital economy in terms of labor resource allocation.

2.The literature review section has already covered the forms of the labor market under the digital economy and the research on the impact of the digital economy on labor allocation. As for the "positive and negative impacts" mentioned by the reviewers, they have also been addressed in the literature review. However, this paper does not intend to structure the literature review as a combination of "positive and negative impacts" because the development of the digital economy is a prevailing trend, and discussing these aspects does not hold much significance.

3. The conclusion that ' These new features not only change labor relations but also profoundly affect the subjective perception of laborers, and reconstruct the evaluation system of labor resource allocation.' is derived from the research conducted by Chen Xiaohong et al. (2022). The data-driven nature of the digital economy is characterized by the efficient presentation and dissemination of massive amounts of information, with data resources becoming a key driver of labor resource allocation. However, it can also create the problem of the digital divide…… cultivates a system of mass innovation and entrepreneurship, achieving flexible employment for many workers." These aspects originate from the background reality in China, and they are observable phenomena, whether it be professional job search websites or popular platforms such as Douyin and Kuaishou for short videos. Therefore, there is no need to specifically seek literature support for these statements.

4. At present, there are no available papers that specifically focus on researching laborers' subjective evaluations in relation to the digital economy. Therefore, it is currently difficult to make direct comparisons with peer-reviewed studies in the empirical research section.

5. Note: (The values in parentheses represent clustered robust standard errors. This applies to all subsequent mentions as well.) have been added in the table.

6. The authors proposed several measures, including strengthening the construction of digital infrastructure, to ensure that all workers can access high-quality internet and information services. They also emphasized the need to reevaluate and understand the societal value of algorithms and to enhance workers' autonomy and initiative in their interactions with algorithmic logic.

Regarding the heterogeneity issue among specific groups, the paper presented specific measures in the conclusion.

Of course, this study only focused on the efficiency of labor resource allocation in the early stage of China's digital economy. With the development of China's digital economy, we will continue to pay attention to this issue and observe whether the conclusions change with the advancement of the digital economy.

7. There is a limited amount of literature from foreign countries that focuses on labor resource allocation issues under the digital economy in China. As for the abundance of Chinese literature, it is mainly due to domestic scholars being the main contributors in researching labor resource allocation in China. It is challenging to find more valuable information to support the arguments of this paper in foreign literature.

8. This point has been revised accordingly, and appropriate annotations have been made in the text.

Response to reviewer #2:

1. The title has been reviewed.

2. M1 is a contrarian indicatorreverse indicator, with higher DM1 values indicating that "the respondent perceives their own socioeconomic status to be relatively lower in the local area."

M2 is a contrarian indicator, with higher M2 value indicating that " a decline in your standard of living compared to five years ago."

M3 is a positive indicator, and a higher value of M3 indicates "a higher frequency of experiencing positive emotions and enjoyment during work."

3. D1, D2, and D3 have been revised to M1, M2, and M3.

4. The relevant literature has been included

5. The authors proposed several measures, including strengthening the construction of digital infrastructure, to ensure that all workers can access high-quality internet and information services. They also emphasized the need to reevaluate and understand the societal value of algorithms and to enhance workers' autonomy and initiative in their interactions with algorithmic logic.

Regarding the heterogeneity issue among specific groups, the paper presented specific measures in the conclusion.

Of course, this study only focused on the efficiency of labor resource allocation in the early stage of China's digital economy. With the development of China's digital economy, we will continue to pay attention to this issue and observe whether the conclusions change with the advancement of the digital economy.

6. The author has made adjustments to the tables in the manuscript.
