# Supplementary material for: Research on the impact of digital economy on labor resource allocation: Evidence from China
Source: PLoS One. 2024 Apr 17;19(4):e0297449. doi: 10.1371/journal.pone.0297449 (PMC11023257; doi:10.1371/journal.pone.0297449)
Supplement: Attachment — Submitted filename: Response to Reviewers.docx [file pone.0297449.s005.docx]

Response to Reviewer #3:

1.The motivation behind this study is the need for responsible innovation and research in the existing labor factor market in the face of the risk challenges posed by the digital economy. This is essential to address the impact of digital elements on the labor force.

2.In the abstract, we have included references to the survivorship bias effect, the digital divide, and algorithmic dominance, all of which can affect the efficiency of labor market allocation in the context of the digital economy.

3.There are traces of my personal writing style in the literature review, and I hope the reviewer can understand and accommodate this.

4.Currently, there is a limited research foundation in this area, especially concerning subjective evaluations of early workers in the digital economy. Consequently, I couldn't find the gaps mentioned by the reviewer.

5.I do not consider the conclusions of this study to be weak. The policy implications have already been reflected in the conclusion.

6.I have conducted a comprehensive review and correction of the grammar in this paper.
