# Supplementary material for: Research on the impact of digital economy on labor resource allocation: Evidence from China
Source: PLoS One. 2024 Apr 17;19(4):e0297449. doi: 10.1371/journal.pone.0297449 (PMC11023257; doi:10.1371/journal.pone.0297449)
Supplement: Attachment — Submitted filename: Response to Reviewers.docx [file pone.0297449.s006.docx]

Response to Reviewer #3:

1. This study examines the impact of the digital economy on the allocation of labor resources in China, using the 2013 CSS survey database as the research sample. Therefore, the conclusions drawn from this study cannot be arbitrarily extended in their applicability. Different countries in different regions have varying digital infrastructures, making it difficult to derive globally applicable conclusions due to differences in cultural, economic, and other factors. For instance, the differentiated research on property quantity and non-unmarried groups carries specific implications within the Chinese economy, making it challenging for the author to forcefully generalize these conclusions.

2. Due to the limited number of papers directly exploring the relationship between the digital economy and the allocation of labor resources, the author has made utmost efforts to compile existing literature. Consequently, it is challenging to further expand the literature on this topic.

3. Regarding suggestions for the future, as mentioned in the conclusion section, " it is essential to consider the subjective perceptions of specific societal groups, and when deemed necessary, implement targeted social relief measures to address their unique needs and challenges."
